# Association between mitochondrial genetic variation and breast cancer risk: The Multiethnic Cohort

**DOI:** 10.1371/journal.pone.0222284

**Published:** 2019-10-02

**Authors:** Yuqing Li, Elena E. Giorgi, Kenneth B. Beckman, Christian Caberto, Remi Kazma, Annette Lum-Jones, Christopher A. Haiman, Loïc Le Marchand, Daniel O. Stram, Richa Saxena, Iona Cheng

**Affiliations:** 1 Department of Epidemiology and Biostatistics, School of Medicine, University of California, San Francisco, California, United States of America; 2 Theoretical Biology and Biophysics, Los Alamos National Laboratory, Los Alamos, New Mexico; 3 University of Minnesota Genomics Center, Minneapolis, Minnesota, United States of America; 4 Epidemiology Program, University of Hawaii Cancer Center, University of Hawaii, Honolulu, Hawaii, United States of America; 5 Roche Pharmaceutical Research and Early Development, Roche Innovation Center Basel, Switzerland; 6 Department of Preventive Medicine, Keck School of Medicine, University of Southern California, Los Angeles, California, United States of America; 7 Center for Human Genetic Research, Department of Anesthesia, Critical Care and Pain Medicine, Massachusetts General Hospital, Boston, Massachusetts, United States of America; 8 Program of Medical and Population Genetics, The Broad Institute of Harvard and MIT, Cambridge, Massachusetts, United States of America; Ohio State University Wexner Medical Center, UNITED STATES

## Abstract

**Background:**

The mitochondrial genome encodes for thirty-seven proteins, among them thirteen are essential for the oxidative phosphorylation (OXPHOS) system. Inherited variation in mitochondrial genes may influence cancer development through changes in mitochondrial proteins, altering the OXPHOS process and promoting the production of reactive oxidative species.

**Methods:**

To investigate the association between mitochondrial genetic variation and breast cancer risk, we tested 314 mitochondrial SNPs (mtSNPs), capturing four complexes of the mitochondrial OXPHOS pathway and mtSNP groupings for rRNA and tRNA, in 2,723 breast cancer cases and 3,260 controls from the Multiethnic Cohort Study.

**Results:**

We examined the collective set of 314 mtSNPs as well as subsets of mtSNPs grouped by mitochondrial OXPHOS pathway, complexes, and genes, using the sequence kernel association test and adjusting for age, sex, and principal components of global ancestry. We also tested haplogroup associations using unconditional logistic regression and adjusting for the same covariates. Stratified analyses were conducted by self-reported maternal race/ethnicity. No significant mitochondrial OXPHOS pathway, gene, and haplogroup associations were observed in African Americans, Asian Americans, Latinos, and Native Hawaiians. In European Americans, a global test of all genetic variants of the mitochondrial genome identified an association with breast cancer risk (P = 0.017, q = 0.102). In mtSNP-subset analysis, the gene *MT-CO2* (P = 0.001, q = 0.09) in Complex IV (cytochrome c oxidase) and *MT-ND2* (P = 0.004, q = 0.19) in Complex I (NADH dehydrogenase (ubiquinone)) were significantly associated with breast cancer risk.

**Conclusions:**

In summary, our findings suggest that collective mitochondrial genetic variation and particularly in the *MT-CO2* and *MT-ND2* may play a role in breast cancer risk among European Americans. Further replication is warranted in larger populations and future studies should evaluate the contribution of mitochondrial proteins encoded by both the nuclear and mitochondrial genomes to breast cancer risk.

## Background

Breast cancer is the most common cancer in women in the United States. In 2019, it has been estimated there will be 271,270 new cases of breast cancer in the United States [1]. Twin studies and familial studies suggested that heredity may account for ~49% of the risk of familial breast cancer [2–4]. To date, over 168 breast cancer risk loci have been identified by genome-wide association studies (GWAS) of breast cancer [5–22]; yet, inherited variants of germline mitochondrial DNA (mtDNA) have not been fully examined in relation to breast cancer susceptibility. To our knowledge, no study to date has comprehensively examined the relationship between the mitochondrial genome variants and breast cancer risk in multiple ethnic populations.

The mitochondrial genome is a small circular DNA molecule that spans 16.6 kb and has no intronic sequences [23]. This genome has become extremely specialized for the synthesis of proteins essential for the oxidative phosphorylation (OXPHOS) system and has retained only a small number of genes over the course of evolution [24]. Thirty-seven genes are encoded by the mitochondrial genome, comprising 13 essential polypeptides of the oxidation phosphorylation system and the RNA machinery necessary for their translation (2 ribosomal RNAs and 22 transfer RNAs)[24]. The remaining proteins of the mitochondrial electron transport chain and those needed for mtDNA maintenance are encoded by nuclear DNA and synthesized by cytoplasmic ribosomes [24].

The primary function of the mitochondrion is the production of the energy molecule, adenosine triphosphate (ATP), through the metabolic pathway of OXPHOS. In addition, the mitochondrion also serves as the major source of reactive oxygen species (ROS). ROS are involved in many cellular processes such as apoptosis, inflammation and oxidative stress. They are generally considered as toxic agents that contribute to aging, a wide variety of degenerative diseases, and cancer [25, 26]. Moreover, the mitochondrion itself is a sensitive target for the damaging effects of ROS. In particular, mtDNA is highly susceptible to oxidative damage due to its lack of protective histones and its close proximity to the electron transport chain, leading to instability of the mitochondrial genome in cancer cells [27–29]. In addition, less efficient DNA repair processes have been reported for mtDNA in comparison to nuclear DNA [30–32].

Variations in mtDNA have the potential to alter mitochondrial function and lead to increased oxidative stress and breast cancer risk [33–39]. Canter et al. tested the association between mtDNA G10398A and breast cancer risk firstly in 48 African American breast cancer cases and 54 African American controls followed by a validation study of 654 breast cancer cases and 605 controls [33], and reported an association in African-Americans (OR = 1.60, p value = 0.013), yet no association in Whites (OR = 1.03, p value = 0.81). Setiawan et al. found no association with mtDNA, G10398A and breast cancer risk [40] in a study of 1,456 African American breast cancer cases and 978 African American controls. Similarly, no association with mtDNA G10398A and breast cancer risk was found in a study of 716 cases and 724 controls in a South Indian population [35]. Recently, Blein et al. conducted a large scale study of 11,421 breast cancer affected and 10,793 unaffected BRCA1/2 mutation carriers of European ancestry, and identified an inverse association between mtDNA haplogroup T1a1 and breast cancer risk (Hazard Ratio = 0.62, 95% Confidence Interval = 0.40–0.95; P = 0.03) [37]. Other small studies have reported other candidate polymorphisms (G9055A, T16519 or G6267A) to be associated with breast cancer risk among European ancestry women [41, 42]. These inconsistent findings may be due to differences in study design, study populations, and small sample sizes with limited statistical power.

To investigate the association between mitochondrial genetic variation and breast cancer risk across multiple racial/ethnic groups, we tested 314 mtSNPs among 5,983 subjects (2,723 breast cancer cases and 3,260 controls) in the Multiethnic Cohort Study (MEC).

## Methods

### Study subjects

Our study included 2,723 incident breast cancer cases and 3,260 controls nested within the MEC, a large population-based cohort of more than 215,000 subjects comprised of African Americans, European Americans, Japanese Americans, Latinos, and Native Hawaiians, who were recruited from 1993 through 1996 at the ages of 45 and 75 years [43]. Blood samples were collected from incident breast and colorectal cancer cases after their diagnosis, as well as a random sample of cohort members to serve as controls from 1996 through 2001, and prospectively from all willing surviving participants from 2002 through 2007. Incident breast cancer cases were identified through cohort linkage to population-based cancer Surveillance, Epidemiology and End Results (SEER) registries in California and Hawaii up to December 9, 2010. Control subjects were women selected to not have breast cancer before cohort entry or during follow-up as of December 9, 2010 and served as matched controls on age (5-year age groups) and race/ethnicity for our nested breast and colorectal cancer studies. This study was approved by the institutional review board at the University of Hawaii and University of Southern California. Written informed consent was obtained from all subjects.

### mtSNPs genotyping

Mitochondrial SNPs were genotyped using the Sequenom platform (n = 186 mtSNPs) [44] and the Illumina Exome array [45] (n = 240 mtSNPs). The 2,723 cases and 3,260 controls were shared between the two genotyping platforms with an average call rate of 99.83%. Forty mtSNPs were common in both genotyping platforms with a concordance rate of 99.3%. Of the 386 unique mtSNPs across the two genotyping platforms, we excluded 72 mtSNPs with MAF<0.1% among the overall sample, resulting in 314 mtSNPs that were distributed across 13 mtDNA genes, comprising four complexes of the OXPHOS pathway, and the tRNA and rRNA subunits (19 mtSNPs per kb).

### Statistical analysis

For the mitochondrial genome, pathway, complex, gene-based analysis, we used the sequence kernel association test (SKAT) [46, 47]. The SKAT_commonrare test is an omnibus procedure allowing for both rare and common variants to contribute to the overall test statistic. For this test, the minor allele frequency of threshold for rare variants was determined by sample size ($T=\frac{1}{\sqrt{2n}}$) [47] (0.1%< MAF<1% for African, Asian, European, Latino ancestry groups; 1%<MAF<5% for Native Hawaiians). All analyses were adjusted for age, self-reported maternal race/ethnicity, and the first five principal components of genetic ancestry. The principal components of genetic ancestry were estimated from a panel of 128 ancestry informative markers [48, 49]. For single mtSNP and haplogroup analyses, we conducted unconditional logistic regression, adjusting for the same covariates listed above. Haplogroups were estimated using the HaploGrep software (http://www.haplogrep.uibk.ac.at) and based on Phylotree build 16 [50, 51]. Additional adjustment for family history of breast cancer, age at menarche, age at first birth, age at menopause, parity, hormone replacement therapy (HRT) use, body mass index (BMI), alcohol, and smoking did not notably alter the results. Thus, these covariates were not included in our final multivariate models. Stratified analyses were conducted by self-reported maternal race/ethnicity. All statistical tests presented are two-sided. A false discovery rate (FDR) was used to account for multiple hypothesis testing for the mitochondrial genome, OXPHOS pathway, complexes, genes, and haplogroup for the five racial/ethnic groups and overall. A false discovery rate q value [52] of 0.20 was used as a threshold to determine statistical significance, which is equivalent to a nominal p value of 6.7x10^-4^ (0.2/300) for ~300 mtSNPs tested and p-value = 0.01 (0.2/20) for the ~20 mitochondrial gene-based tests.

## Results

Study characteristics of the 5,983 study subjects (2,723 breast cancer cases; 3,260 controls) are presented in Table 1. The distribution of maternal race/ethnicity was 23.3% African Americans, 28.7% Asian Americans, 25.5% European Americans, 16.4% Latinos and 6.1% Native Hawaiians. Breast cancer cases were of younger age at menarche, older age at menopause, had fewer children, higher BMI, had higher use of hormone replacement therapy (HRT), more likely to have a family history of breast cancer, consume alcohol, and have a history of diabetes than controls. Approximately 72.2% of breast cancer cases had localized disease and 72.1% were hormone receptor positive. S1 Table shows the distribution of study characteristics by maternal race/ethnicity.

**Table 1:** Study characteristics of 2,723 breast cancer cases and 3,260 controls in the MEC.

Characteristics	Categories	Cases (n = 2,723)	Controls (n = 3,260)
Age†, mean (SD)		66.10 (0.17)	66.25 (0.16)
Maternal race/ethncity, n(%)††	African Americans	463 (17.07%)	923 (28.43%)
	Asian Americans	808 (29.78%)	900 (27.73%)
	Latinos	512 (18.87%)	464 (14.29%)
	European Americans	745 (27.46%)	769 (23.69%)
	Native Hawaiians	179 (6.6%)	186 (5.73%)
Age at Menarche	Younger than 13yrs	1437 (53.62%)	1590 (49.3%)
	Age between 13~14yrs	960 (35.82%)	1267 (39.29%)
	Age over than 15yrs	283 (10.56%)	368 (11.41%)
Age at first birth	Nulliparis	408 (15.37%)	379 (11.87%)
	Younger than 20yrs	608 (22.9%)	873 (27.34%)
	Age between 21~30yrs	1420 (53.48%)	1698 (53.18%)
	Age over than 30yrs	219 (8.25%)	243 (7.61%)
Age at menopause††	Pre-menopausal	162 (11.83%)	203 (13.22%)
	natural menopause at age <45	397 (29%)	483 (31.45%)
	natural menopause at age 45–49	621 (45.36%)	674 (43.88%)
	natural menopause at age 50–54	189 (13.81%)	176 (11.46%)
Parity	Nulliparous	417 (15.42%)	396 (12.2%)
	1 Child	312 (11.54%)	354 (10.91%)
	2~3 children	1244 (46.01%)	1533 (47.23%)
	More than 4 children	731 (27.03%)	963 (29.67%)
HRT use	Never used HRT	1114 (41.8%)	1505 (47.1%)
	Ever used estrogen or progestin	463 (17.37%)	602 (18.84%)
	Current use estrogen or progestin	1088 (40.83%)	1088 (34.05%)
BMI, n (%)	<25 kg/m2	946 (39.66%)	1213 (42.35%)
	> = 25 kg/m2	1439 (60.34%)	1651 (57.65%)
Family history of breast cancer, n (%)	Yes	447 (16.042%)	382 (11.72%)
Alcohol, g/day, mean (SD)	Yes	5.58 (16.35)	4.51 (14.55)
Smoking, pack/year, mean (SD)	Yes	7.35 (12.69)	6.85 (12.22)
History of Diabetes, n (%)	Yes	317 (12.44%)	319 (10.38%)
stage (%)*	Localized	1951 (72.15%)	--
	Advanced*	726 (26.78%)	--
HR status‡	Positive	1953 (72.07%)	--

†Age of diagnosis for cases and age at blood draw for controls

††Due to missing value, the total doesn't add up to 100%.

‡Either estrogen Receptor (ER) or progesterone Receptor (PR) status is positive.

*Including regional and distant disease.

### Mitochondrial genome, pathway, and gene analysis

No statistically significant associations were observed with the mitochondrial genomes, pathways, or genes in all groups combined and among African Americans, Asian Americans, Latinos, and Native Hawaiians (Table 2). Yet, in European Americans, a global test of the mitochondrial genome (collective set of 261 mtSNPs with MAF >0.1%) revealed an association with breast cancer risk (P = 0.017, q = 0.102; Table 2). In addition, two mtDNA genes, NADH dehydrogenase 2 (*MT-ND2*; P = 0.004, q = 0.19) and cytochrome c oxidase II (*MT-CO2*; P = 0.001, q = 0.095) were associated with breast cancer risk in European Americans (Table 2).

**Table 2:** Association between mitochondrial genome, pathways, genes and breast cancer risk by maternal race/ethnicity and overall in the MEC.

Maternal Race/Ethnicity	Mitochondrial genome			Mitochondrial Oxidative phosphoralation pathway			Mitochondrial complex				Mitochondrial gene				
	SNPs	P value	q value	SNPs	P value*	q value	Complex	SNPs	P value*	q value	Gene	SNPs	No. SNPs with MAF> = 0.01^#^	P value*	q value
**African Americans**	290	0.752	0.902	208	0.743	0.968	Complex I	120	0.613	0.993	*MT-ND1*	17	6	0.066	0.523
											*MT-ND2*	21	7	0.745	1.000
											*MT-ND3*	8	5	0.863	1.000
											*MT-ND4*	20	9	0.344	1.000
											*MT-ND4L*	4	2	0.608	1.000
											*MT-ND5*	41	19	0.970	1.000
											*MT-ND6*	9	4	0.703	1.000
							Complex III	37	0.545	0.993	*MT-CYB*	37	14	0.545	1.000
							Complex IV	37	0.988	0.993	*MT-CO1*	20	11	0.978	1.000
											*MT-CO2*	5	5	1.000	1.000
											*MT-CO3*	12	7	0.726	1.000
							Complex V	14	0.542	0.993	*MT-ATP6*	10	4	0.407	1.000
											*MT-ATP8*	4	1	0.791	1.000
											*MT-DLOOP*	6	3	0.544	1.000
											rRNA	46	22	0.770	1.000
											tRNA	30	10	0.751	1.000
**Asian Americans**	193	0.972	0.972	146	0.923	0.968	Complex I	79	0.653	0.993	*MT-ND1*	12	3	0.203	0.891
											*MT-ND2*	14	14	0.459	1.000
											*MT-ND3*	5	3	0.235	0.891
											*MT-ND4*	18	3	0.461	1.000
											*MT-ND4L*	2	1	0.843	1.000
											*MT-ND5*	23	10	0.944	1.000
											*MT-ND6*	5	2	0.904	1.000
							Complex III	31	0.974	0.993	*MT-CYB*	31	13	0.954	1.000
							Complex IV	22	0.993	0.993	*MT-CO1*	9	2	0.980	1.000
											*MT-CO2*	4	2	0.882	1.000
											*MT-CO3*	9	4	0.599	1.000
							Complex V	14	0.320	0.993	*MT-ATP6*	11	5	0.272	0.891
											*MT-ATP8*	3	1	0.974	1.000
											MT-DLOOP	5	2	0.968	1.000
											rRNA	24	13	0.945	1.000
											tRNA	18	3	0.888	1.000
**Latinos**	248	0.384	0.816	185	0.457	0.968	Complex I	107	0.489	0.993	*MT-ND1*	15	4	0.758	1.000
											*MT-ND2*	16	6	0.336	1.000
											*MT-ND3*	7	3	0.756	1.000
											*MT-ND4*	20	7	0.257	0.891
											*MT-ND4L*	4	0	0.080	0.585
											*MT-ND5*	36	6	0.588	1.000
											*MT-ND6*	9	3	0.846	1.000
							Complex III	32	0.690	0.993	*MT-CYB*	32	5	0.684	1.000
							Complex IV	32	0.413	0.993	*MT-CO1*	17	4	0.447	1.000
											*MT-CO2*	5	1	0.204	0.891
											*MT-CO3*	10	3	0.562	1.000
							Complex V	14	0.170	0.714	*MT-ATP6*	9	2	0.155	0.775
											*MT-ATP8*	5	1	0.239	0.891
											MT-DLOOP	5	2	0.855	1.000
											rRNA	41	11	0.112	0.709
											tRNA	17	2	0.917	1.000
**European Americans**	**261**	**0.017**	**0.102**	189	0.015	0.252	Complex I	105	0.022	0.420	*MT-ND1*	14	3	0.605	1.000
											***MT-ND2***	**17**	**5**	**0.004**	**0.190**
											*MT-ND3*	7	2	0.042	0.496
											*MT-ND4*	18	8	0.349	1.000
											*MT-ND4L*	3	1	0.222	0.891
											*MT-ND5*	36	8	0.031	0.496
											*MT-ND6*	10	1	0.036	0.496
							Complex III	35	0.047	0.494	*MT-CYB*	35	9	0.047	0.496
							Complex IV	34	0.030	0.420	*MT-CO1*	18	2	0.297	0.941
											***MT-CO2***	**5**	**2**	**0.001**	**0.095**
											*MT-CO3*	11	3	0.363	1.000
							Complex V	15	0.069	0.580	*MT-ATP6*	13	2	0.137	0.766
											*MT-ATP8*	2	0	0.011	0.348
											MT-DLOOP	6	3	0.259	0.891
											rRNA	44	11	0.154	0.775
											tRNA	22	8	0.021	0.496
**Native Hawaiians**	75	0.544	0.816	57	0.785	0.968	Complex I	32	0.859	0.993	*MT-ND1*	4	1	0.563	1.000
											*MT-ND2*	6	2	0.588	1.000
											*MT-ND3*	4	1	0.768	1.000
											*MT-ND4*	6	0	0.520	1.000
											*MT-ND5*	11	1	1.000	1.000
											*MT-ND6*	1	0	0.058	0.501
							Complex III	10	0.504	0.993	*MT-CYB*	10	1	0.510	1.000
							Complex IV	10	0.905	0.993	*MT-CO1*	5	2	0.709	1.000
											MT-CO2	2	0	0.858	1.000
											*MT-CO3*	3	0	0.638	1.000
							Complex V	5	0.492	0.993	*MT-ATP6*	4	1	0.725	1.000
											*MT-ATP8*	1	0	0.058	0.501
											MT-DLOOP	1	0	0.648	1.000
											rRNA	10	3	0.102	0.692
											tRNA	7	1	0.459	1.000
**Overall**	314	0.525	0.816	227	0.537	0.968	Complex I	127	0.380	0.993	*MT-ND1*	17	10	0.590	1.000
											*MT-ND2*	22	19	0.039	0.496
											*MT-ND3*	9	8	0.270	0.891
											*MT-ND4*	23	16	0.515	1.000
											*MT-ND4L*	4	4	0.765	1.000
											*MT-ND5*	43	27	0.741	1.000
											*MT-ND6*	9	8	0.576	1.000
							Complex III	44	0.821	0.993	*MT-CYB*	44	25	0.821	1.000
							Complex IV	38	0.434	0.993	*MT-CO1*	20	15	0.636	1.000
											*MT-CO2*	5	5	0.245	0.891
											*MT-CO3*	13	9	0.465	1.000
							Complex V	18	0.797	0.993	*MT-ATP6*	15	9	0.766	1.000
											*MT-ATP8*	3	2	0.595	1.000
											MT-DLOOP	6	4	0.788	1.000
											rRNA	52	38	0.715	1.000
											tRNA	29	13	0.136	0.766

*Pathway and gene-based analysis were adjusted for age, sex, maternal race/ethnicity (all groups combined), and genetic ancestry.

^#^ Treshold of MAF to differenciate common and rare mtSNPs 0.1%<MAF<1% in each maternal ethnicities exept in Hawaiian 1%<MAF<5%.

### MtSNP and haplogroup associations

**Fig 1:**
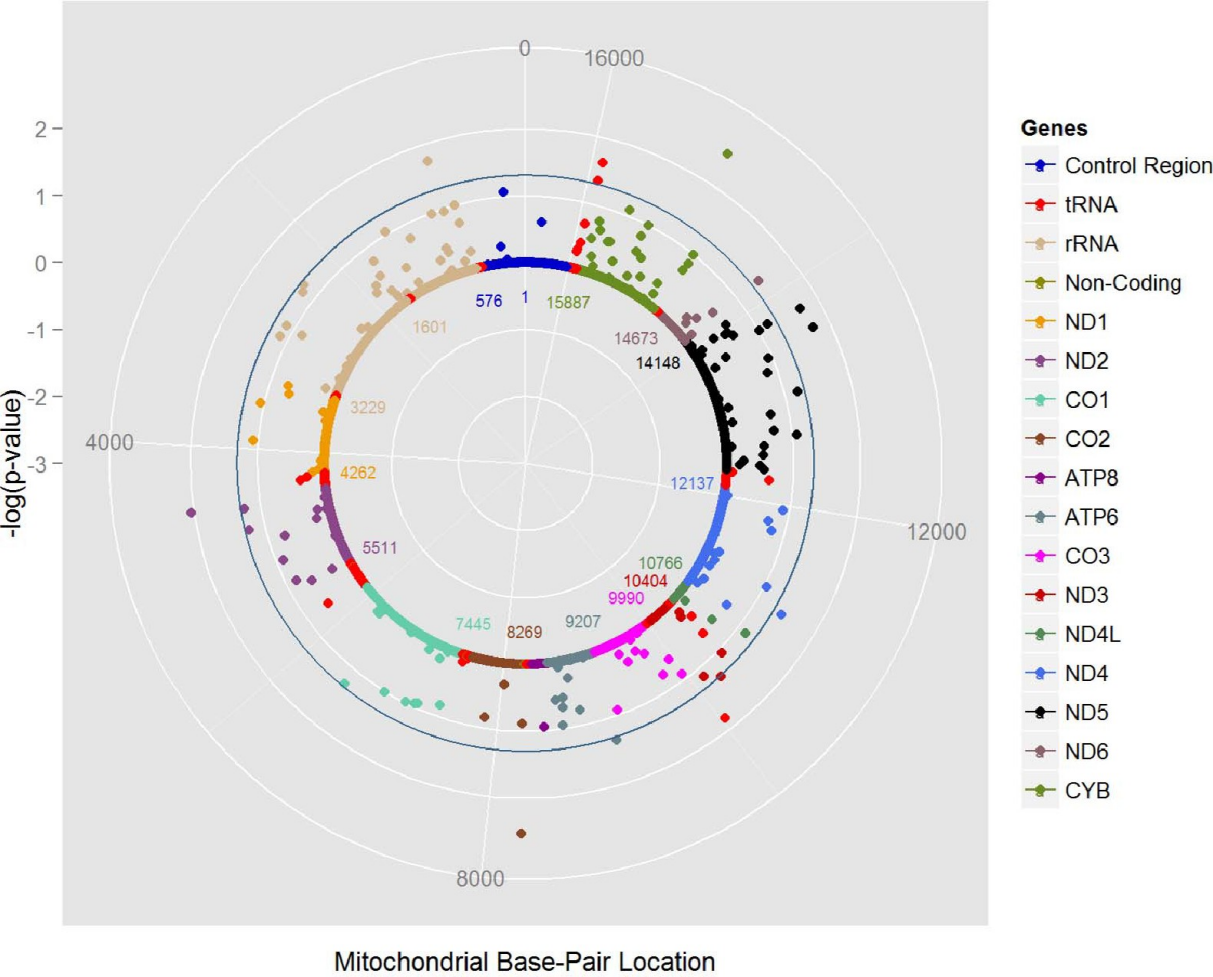
Mitochondrial solar plot for European Americans. The three white circles from the outer to inner circles correspond to the P value of 10^−3^, 10^−2^ and 10^−1^. The teal circle represents a p-value of 0.05. Each dot represents the mtSNP association with breast cancer color coded by mitochondrial gene.

Overall among all groups combined, 11 of 314 mtSNPs were associated with breast cancer risk at P<0.05 (S2 Table) with the most significant association seen with tRNA mtSNP (mt15904; P = 0.005) that did not reach our FDR criterion of statistical significance. In maternal race/ethnicity stratified analyses of mtSNPs (MAF>0.01), 5 of 280 mtSNPs, 4 of 189 mtSNPs, 13 of 249 mtSNPs, and 7 of 240 mtSNPs were associated with breast cancer risk at P<0.05 in African Americans, Asian Americans, European Americans, and Latinos, respectively and did not reach our statistical significance threshold (S2 Table and Fig 1). No mtSNP associations were observed in Native Hawaiians. Haplogroup associations and frequencies by case/control status for each maternal racial/ethnic group are presented in S3 Table. No haplogroup associations were seen across the five racial/ethnic groups.

## Discussion

In this study, we comprehensively examined the association between the mitochondrial genetic variation and breast cancer risk in five racial/ethnic groups. To our knowledge, this is the first study to examine the collective mitochondrial genome, pathways, genes, and mtSNPs for their associations with breast cancer risk in a multiethnic population. No mitochondrial genome associations were observed in African Americans, Asian Americans, Latinos, and Native Hawaiians. Yet, an association between mitochondrial genetic variation and breast cancer risk was observed among European Americans.

In European Americans, the most significant association was seen with the *MT-CO2* gene that is part of the respiratory Complex IV (cytochrome c oxidase). In vitro studies have reported over expression of *MT-CO2* in human breast cancer epithelial cells and reduced breast tumor growth with treatment of a *MT-CO2* inhibitor [53, 54]. The other significant association was observed with the *MT-ND2* gene, a member of the OXPHOS pathway that encodes for the subunit of NADH. It is reasonable to hypothesize that mtDNA coding variants in *MT-CO2* and *MT-ND2* genes may have cis-acting effects on the expression of these genes that may be distinct to European ancestry populations as a recent study reported specific SNP-gene expression patterns between African and Caucasian populations [55]. In addition, nuclear DNA encoded transcription factors have been shown to bind to coding regions in mtDNA to regulate gene expression [56–58].

In a large study of *BRCA1/2* mutation carriers in the CIMBA consortium (11,421 cases/ 10,793 controls), haplogroup T1a1 was inversely associated with breast cancer risk (Hazard Ratio = 0.62; P = 0.03) among *BRCA1* carriers of European ancestry [37]. Our study did not observe an association between haplogroup T1a1 and breast cancer risk, which may be attributed to differences in our population-based study population in comparison to *BRCA1/2* carriers and differences in study power. A small study of European women (164 breast cancer cases/ 164 controls) reported haplogroup H to be associated with breast cancer risk (OR  =  2.0; 95% CI: 1.1–3.5) [36]. In our study, haplogroup HV was not statistically significantly associated with breast cancer risk in European Americans (OR = 1.59, 95%CI: 0.99–2.57, P = 0.059) (S3 Table).

Two small case-control studies reported mtDNA G13708A to be associated with breast cancer risk in African American and South Indian women, but three other studies [34, 35, 40] failed to confirm an association. We found no association with this specific polymorphism and the *MT-ND5* gene.

Our study has several strengths. Given the wide spectrum of rare, low-frequency, and common genetic variants in the mitochondrial genome, the use of the SKAT common/rare approach to collectively test multiple risk alleles has improved statistical power to detect modest effects than single SNP tests and overcomes the limitation of previous methods that upweight rare variants [47, 59]. Using this approach, we were able to capture the role of multiple mtDNA genes involved in breast cancer risk. In addition, we expect bias due to heteroplasmy to be unlikely, as prior work has reported heteroplasmy in blood DNA to be quite small [60]. We do recognize the limitation of the modest sample size to detect weak genetic effects for certain subgroups such as Native Hawaiians.

## Conclusion

In summary, our findings suggest that collectively multiple mtDNA variants may play a role in breast cancer risk in European Americans. The findings of association between the mitochondrial OXPHOS pathway and multiple mitochondrial complexes and genes warrant replication in other European population. Further studies in larger populations should evaluate both the mitochondrial genome and nuclear encoded mitochondrial genomes to fully examine their contribution to breast cancer risk. A recent study examined the association between nuclear genetic variation and mitochondrial transcript abundance based on genotyping data and RNA sequencing data for 36 tissues/cell types, and found 64 nuclear loci associated with expression level of 14 genes encoded by the mitochondrial genome that included missense variants of genes involved in mitochondrial function [61].

## Supporting information

S1 Table Study characteristics of 2,723 breast cancer cases and 3,260 controls by maternal race/ethnicity in the MEC. (XLSX)

S2 Table 314 mtSNPs tested in 2,723 breast cancer cases and 3,260 controls in the MEC. (XLSX)

S3 Table Association between mitochondrial haplogroups and breast cancer risk by maternal race/ethnicity in the MEC. (XLSX)

## Abbreviations

GWAS: genome-wide association study
OXPHOS: oxidative phosphorylation
mtSNPs: mitochondrial SNPs
mtDNA: mitochondrial DNA
ATP: adenosine triphosphate
ROS: reactive oxygen species
MEC: Multiethnic Cohort Study
SKAT: sequence kernel association test
HRT: hormone replacement therapy
BMI: body mass index
FDR: false discovery rate
MT-ND2: Mitochondrial NADH dehydrogenase 2
MT-CO2: Mitochondrial cytochrome c oxidase II
